# Supplementary material for: Protective Effects of Bromelain in Testicular Torsion-Detorsion: Reducing Inflammation, Oxidative Stress, and Apoptosis While Enhancing Sperm Quality
Source: Biomolecules. 2025 Feb 15;15(2):292. doi: 10.3390/biom15020292 (PMC11852452; doi:10.3390/biom15020292)
Supplement: Supplementary file 1 [file biomolecules-15-00292-s001.zip › biomolecules-3433774-supplementary.pdf]

Western bands raw data

BCL-2

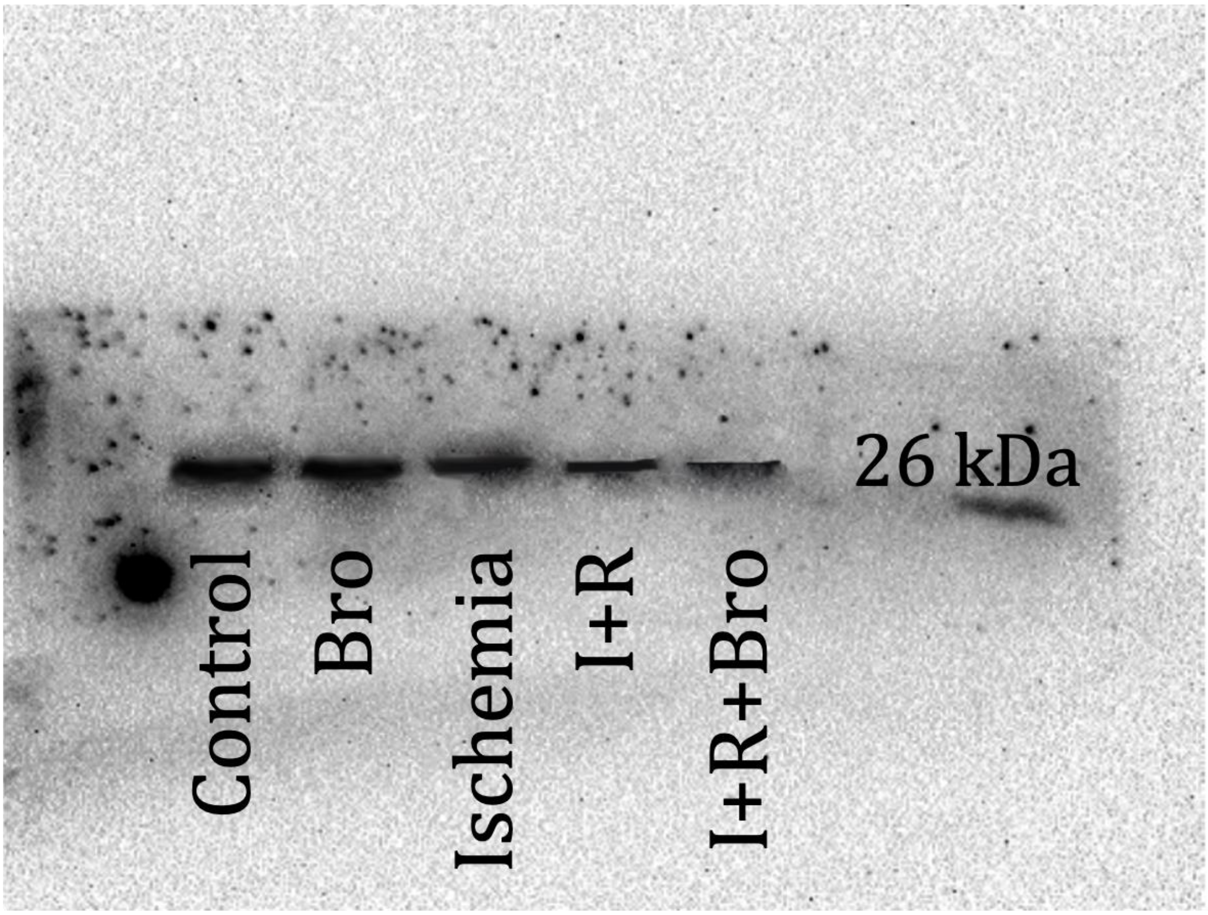

S.No	Groups	Band-1	Band-2	Average	STDEV
1	Control	0.62	0.59	0.605	0.017
2	Bro	0.55	0.57	0.56	0.012
3	Ischemia	0.22	0.32	0.27	0.058
4	I+R	0.18	0.23	0.205	0.029
5	I+R+Bro	0.3	0.4	0.35	0.058

Caspase-3

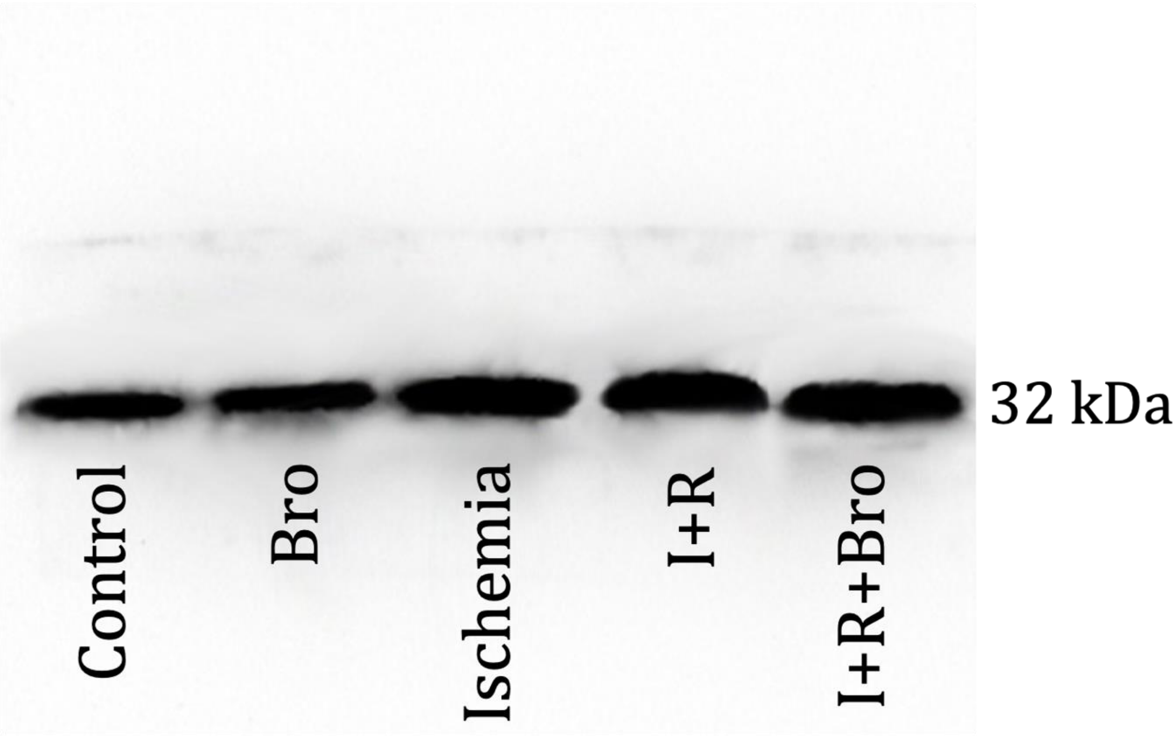

S.No	Groups	Band-1	Band-2	Average	STDEV
1	Control	0.13	0.17	0.15	0.023
2	Bro	0.2	0.24	0.22	0.023
3	Ischemia	0.5	0.7	0.6	0.115
4	I+R	0.54	0.79	0.665	0.144
5	I+R+Bro	0.32	0.44	0.38	0.069

TLR4

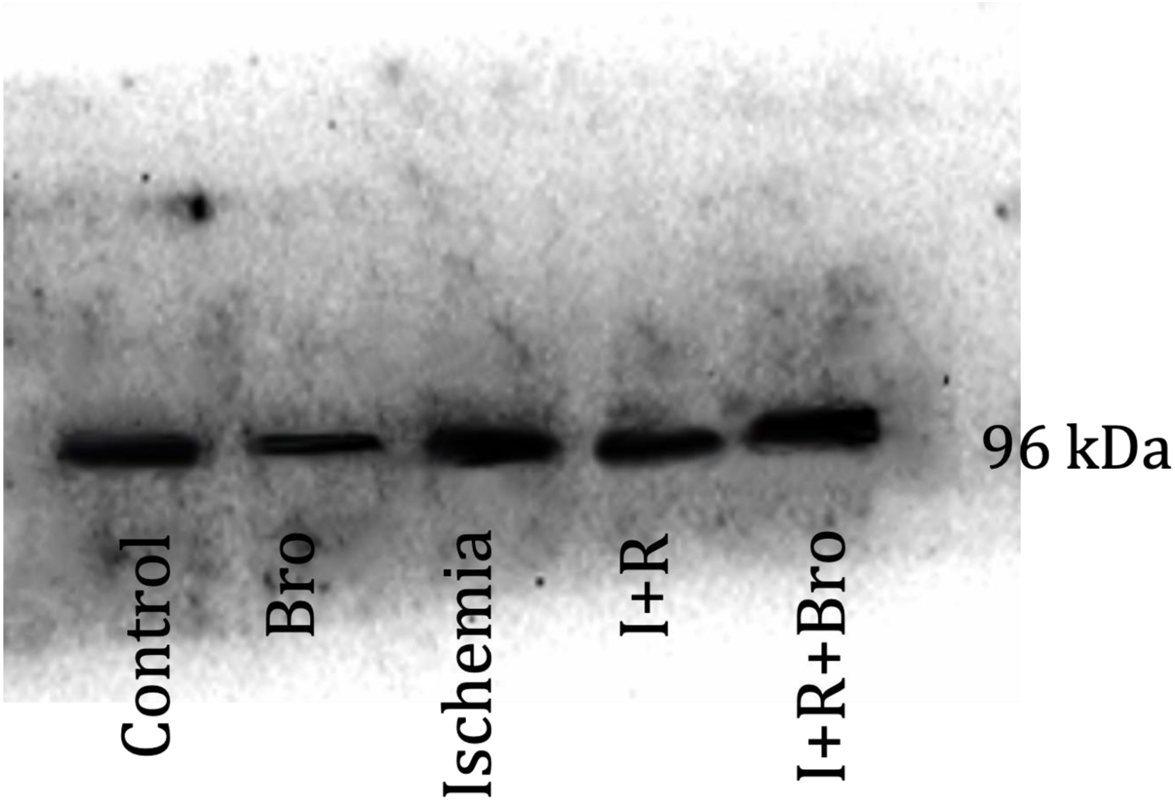

S.No	Groups	Band-1	Band-2	Average	STDEV
1	Control	0.19	0.25	0.22	0.035
2	Bro	0.34	0.25	0.295	0.052
3	Ischemia	0.66	78	39.33	44.652
4	I+R	0.95	0.8	0.875	0.087
5	I+R+Bro	0.46	0.4	0.43	0.035

HO-1

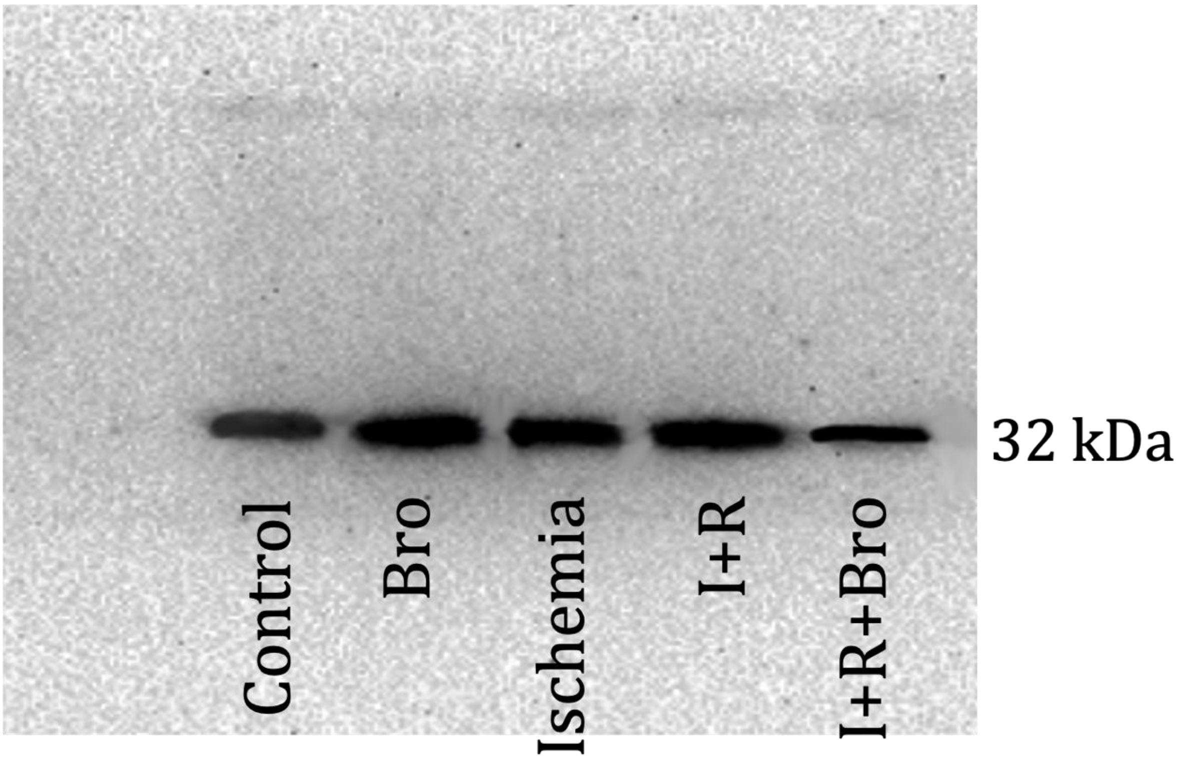

S.No	Groups	Band-1	Band-2	Average	STDEV
1	Control	0.6	0.71	0.655	0.064
2	Bro	0.67	0.55	0.61	0.069
3	Ischemia	0.25	0.34	0.295	0.052
4	I+R	0.22	0.29	0.255	0.040
5	I+R+Bro	0.35	0.42	0.385	0.040

NRF-2

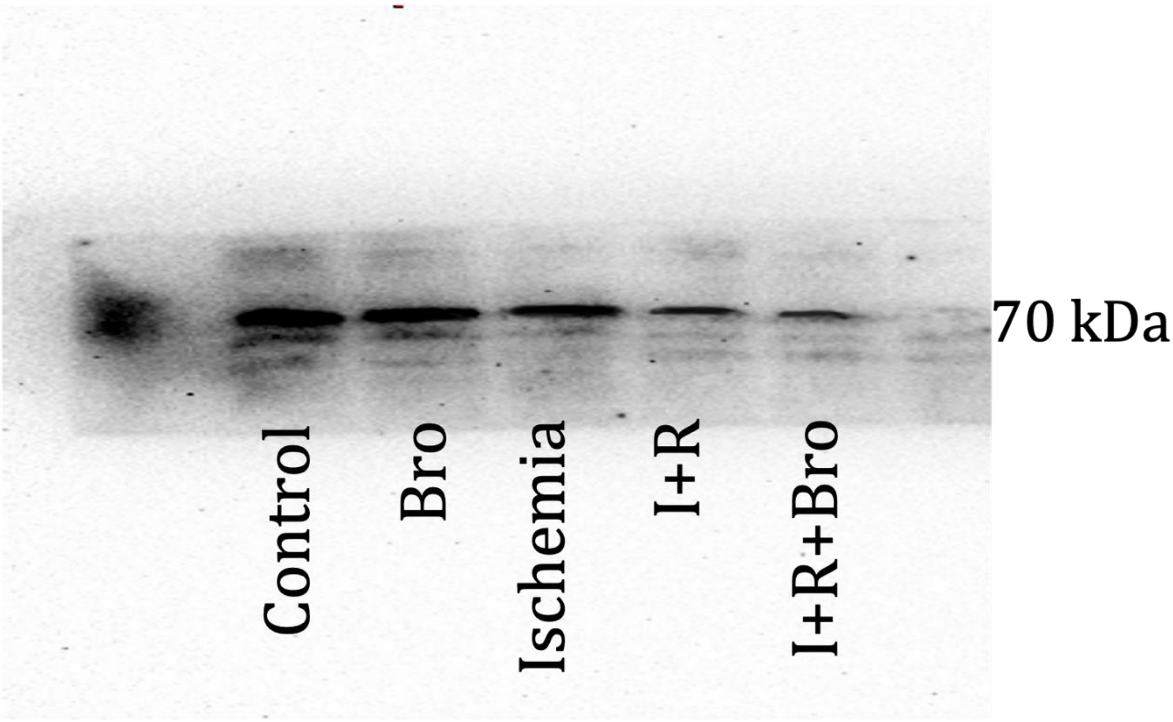

S.No	Groups	Band-1	Band-2	Average	STDEV
1	Control	0.51	0.63	0.57	0.069
2	Bro	0.55	0.48	0.515	0.040
3	Ischemia	0.28	0.35	0.315	0.040
4	I+R	0.22	0.18	0.2	0.023
5	I+R+Bro	0.49	0.41	0.45	0.046

AKT1

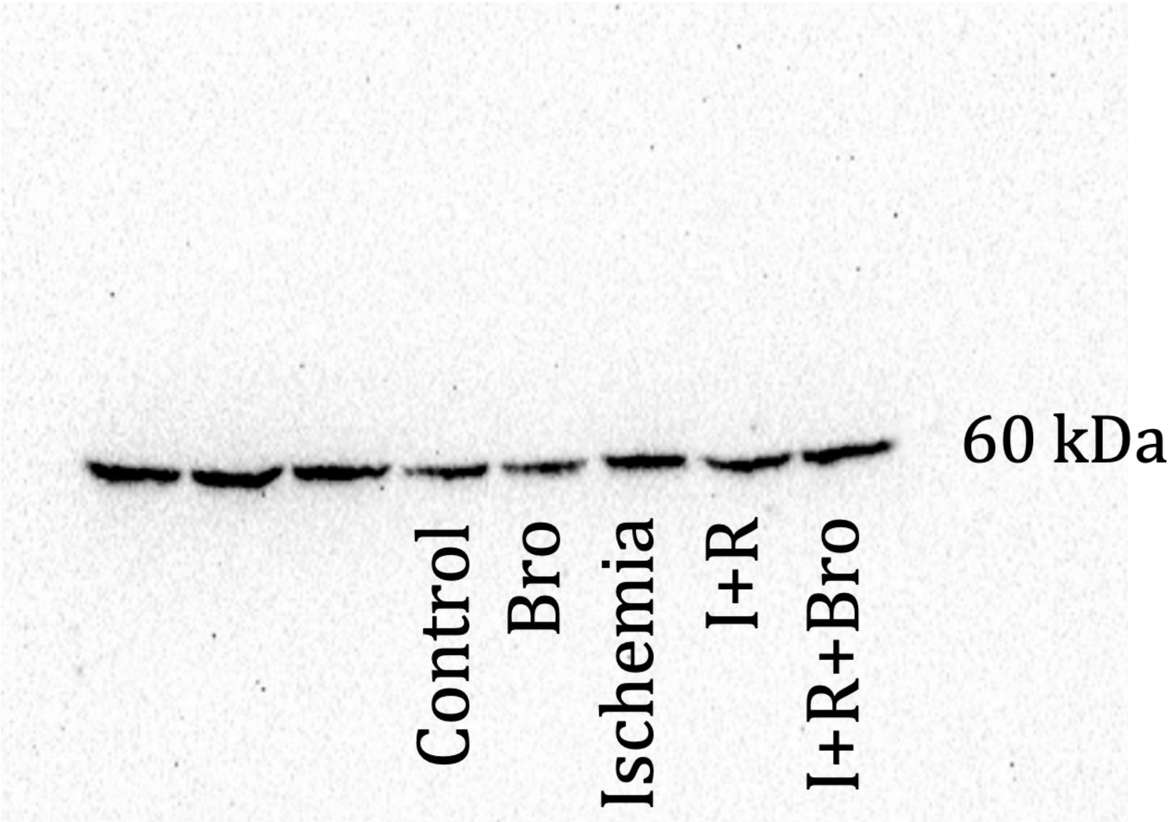

S.No	Groups	Band-1	Band-2	Average	STDEV
1	Control	0.38	0.33	0.355	0.029
2	Bro	0.28	0.4	0.34	0.069
3	Ischemia	0.41	0.46	0.435	0.029
4	I+R	0.55	0.48	0.515	0.040
5	I+R+Bro	0.3	0.37	0.335	0.040

mTOR

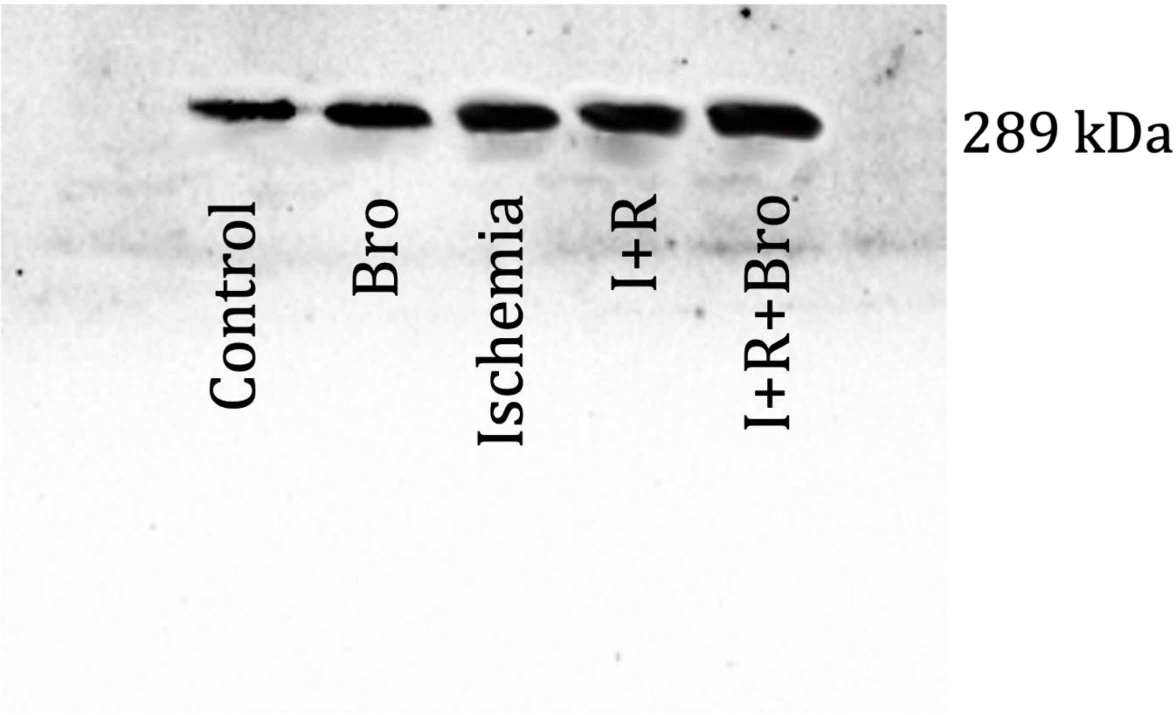

S.No	Groups	Band-1	Band-2	Average	STDEV
1	Control	0.22	0.2	0.21	0.012
2	Bro	0.28	0.25	0.265	0.017
3	Ischemia	0.39	0.42	0.405	0.017
4	I+R	0.4	0.5	0.45	0.058
5	I+R+Bro	0.35	0.4	0.375	0.029

PI3K

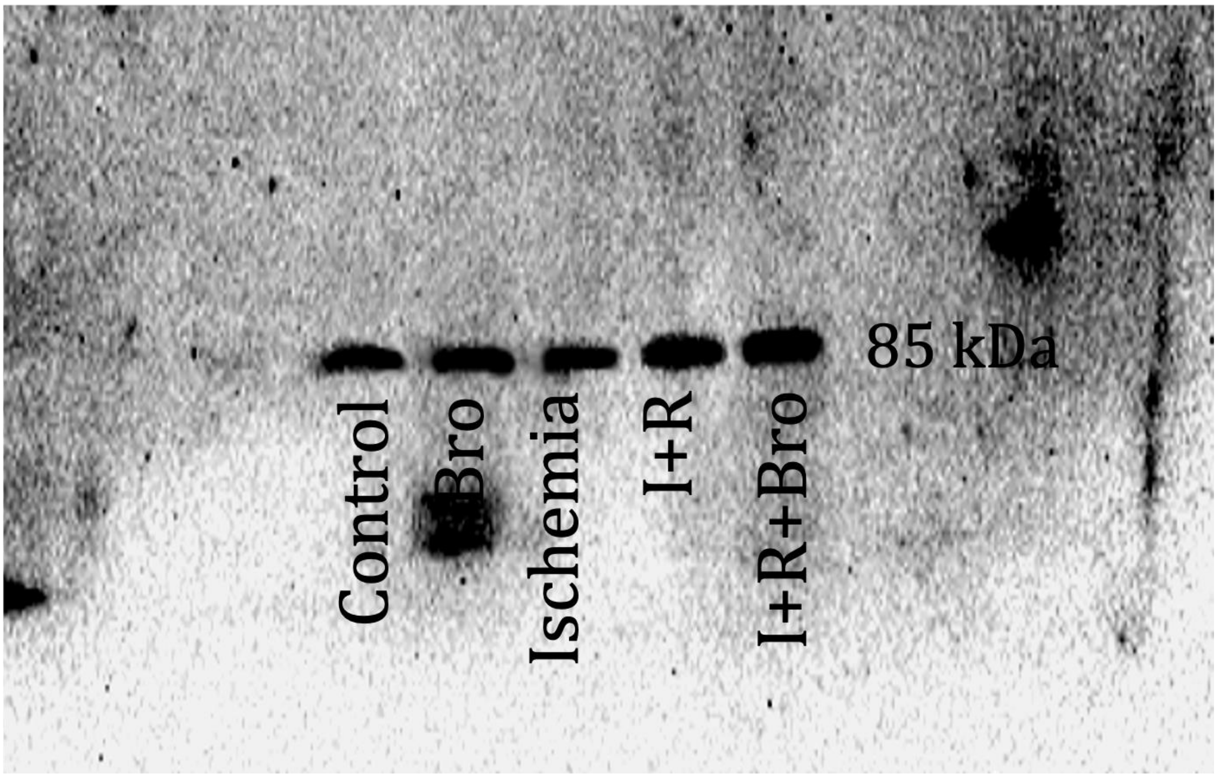

S.No	Groups	Band-1	Band-2	Average	STDEV
1	Control	0.1	0.12	0.11	0.012
2	Bro	0.11	0.15	0.13	0.023
3	Ischemia	0.3	0.34	0.32	0.023
4	I+R	0.42	0.38	0.4	0.023
5	I+R+Bro	0.27	0.3	0.285	0.017

β-Actin

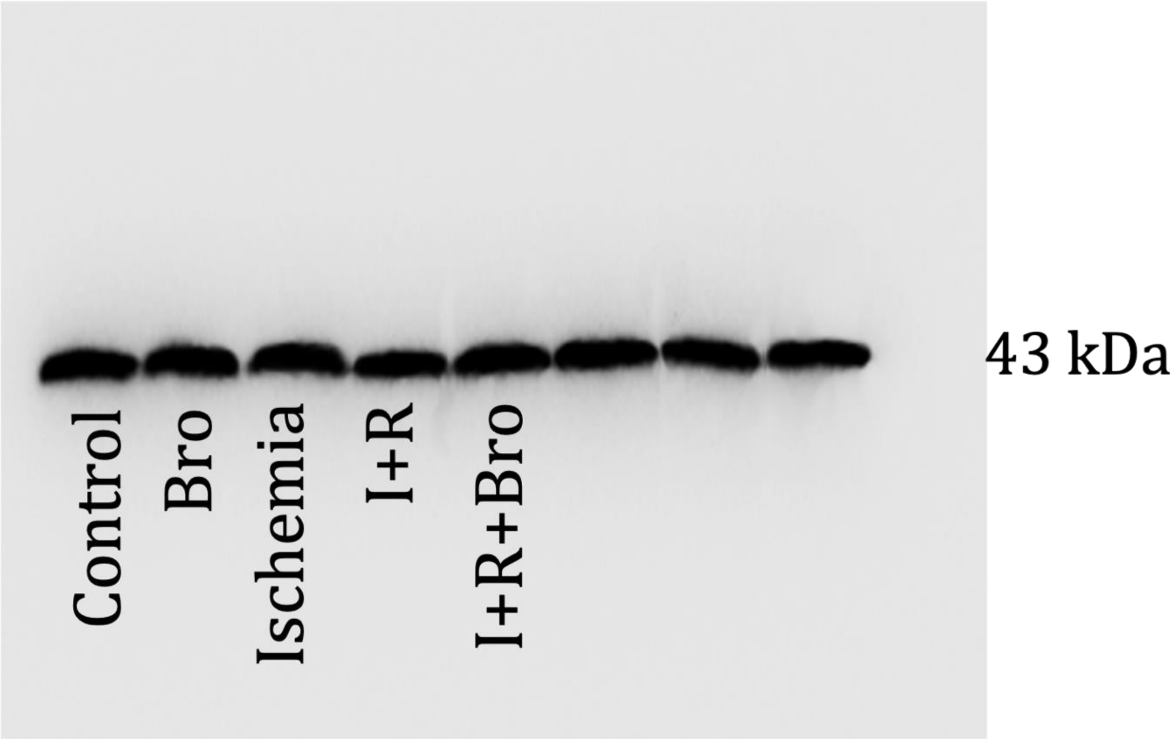

S.No	Groups	Band-1	Band-2	Average	STDEV
1	Control	0.22	0.25	0.235	0.017
2	Bro	0.21	0.26	0.235	0.029
3	Ischemia	0.18	0.23	0.205	0.029
4	I+R	0.2	0.24	0.22	0.023
5	I+R+Bro	0.21	0.26	0.235	0.029
